# Identification of key genes and pathways in atherosclerosis using integrated bioinformatics analysis

**DOI:** 10.1186/s12920-023-01533-8

**Published:** 2023-05-13

**Authors:** Shihuan Li, Suqin Li, Qingjie Li, Qiaofeng Zhou, Wenli Liao, Liangzhu Yu, Changhan Ouyang, Hongli Xia, Chao Liu, Mincai Li

**Affiliations:** 1 Hubei Key Laboratory of Diabetes and Angiopathy, Xianning Medical College, Hubei University of Science and Technology, Xianning, 437100 People’s Republic of China; 2 School of Basic Medical Sciences, Xianning Medical College, Hubei University of Science and Technology, Xianning, 437100 People’s Republic of China; 3 School of Pharmacy, Xianning Medical College, Hubei University of Science and Technology, Xianning, 437100 People’s Republic of China; 4 The Central Hospital of Xianning, Xianning Medical College, Hubei University of Science and Technology, Xianning, 437100 People’s Republic of China

**Keywords:** Atherosclerosis, Differentially expressed genes (DEGs), Network modules, Biomarker, Bioinformatics analysis

## Abstract

**Background:**

Atherosclerosis (AS) is a chronic inflammatory disease that might induce severe cardiovascular events, such as myocardial infarction and cerebral infarction. These risk factors in the pathogenesis of AS remain uncertain and further research is needed. This study aims to explore the potential molecular mechanisms of AS by bioinformatics analyses.

**Methods:**

GSE100927 gene expression profiles, including 69 AS samples and 35 healthy controls, were downloaded from Gene Expression Omnibus database and indenfied for key genes and pathways in AS.

**Results:**

A total of 443 differentially expressed genes (DEGs) between control and AS were identified, including 323 down-regulated genes and 120 up-regulated genes. The Gene ontology terms enriched by the up-regulated DEGs were associated with the regulation of leukocyte activation, endocytic vesicle, and cytokine binding, while the down-regulated DEGs were associated with negative regulation of cell growth, extracellular matrix, and G protein-coupled receptor binding. KEGG pathway analysis showed that the up-regulated DEGs were enriched in Osteoclast differentiation and Phagosome, while the down-regulated DEGs were enriched in vascular smooth muscle contraction and cGMP-PKG signaling pathway. Using the modular analysis of Cytoscape, we identified 3 modules mainly involved in Leishmaniasis and Osteoclast differentiation. The GSEA analysis showed the up-regulated gene sets were enriched in the ribosome, ascorbated metabolism, and propanoate metabolism. The LASSO Cox regression analysis showed the top 3 genes were TNF, CX3CR1, and COL1R1. Finally, we found these immune cells were conferred significantly higher infiltrating density in the AS group.

**Conclusions:**

Our data showed the pathway of Osteoclast differentiation and Leishmaniasis was involved in the AS process and we developed a three-gene model base on the prognosis of AS. These findings clarified the gene regulatory network of AS and may provide a novel target for AS therapy.

**Supplementary Information:**

The online version contains supplementary material available at 10.1186/s12920-023-01533-8.

## Introduction

Atherosclerosis is one of the most common diseases in the cardiovascular system, which threatens human health. The vascular disease causes many vascular complications affecting the cardiovascular system [1, 2]. AS mainly affects the large and medium-sized arteries, and ultimately causes the reduction in arterial inflow or loss of organs, resulting in cells or tissues atrophy/necrosis. As we know, atherosclerotic plaque leads to stenosis and the vulnerability of plaque rupture of a coronary artery, which contributes to the increased risk of cardiovascular events and morbidity and mortality.

The pathological manifestations of AS exhibit the formation of fatty-fibrous plaque, including the adhesion of activated leukocytes, the proliferation of vascular smooth muscle cells (VSMC), and the degradation of the extracellular matrix, angiogenesis, and calcification. Advanced atherosclerotic plaques developed a morphologically fibrous cap composed of VSMCs proliferating and migrating from the vessel wall into the plaque. Our previous studies have reported that pathophysiological factors were involved in the development of AS, including the phenotypic switch of VMSC, the source of oxidative stress, and mitochondrial injury [3, 4].

Atherosclerosis is primarily a chronic inflammatory disease of the arterial wall response to injury, which is mediated by the oxidized low-density lipoprotein (LDL) [5]. In acute artery injury, the endothelial cytokine and the leukocyte adhesion promote the extravasation of monocyte into the sub-endothelial space [6]. Mezentsev reported that desialylation of lipoproteins was an important risk factor in AS and that desialylation of LDL causes the uncontrolled accumulation of lipids by the artery cells [7]. Bezsonov reported that mitochondrial DNA (mtDNA) mutations are associated with AS using leukocytes of atherosclerotic patients [8]. However, the molecular mechanisms involved in the AS process are incompletely understood.

More research will be needed in the public health and medical field and might reduce the incidence and mortality. Given that microarray analysis can provide an enormous amount of data about gene expression, these analysis methods have been used to predict novel prognostic markers and therapeutic targets [9]. In the current study, we downloaded the microarray dataset, GSE100927, from the NCBI-Gene Expression Omnibus database (GEO). The GSE100927 dataset contained 69 AS samples and 35 control samples. Differentially expressed genes (DEGs) were analyzed, and the pathway and Gene ontology enrichment analyses were identified. Furthermore, protein–protein interaction (PPI) networks and modular analysis were constructed to identify hub genes. The module genes were enriched in KEGG pathways. Predictors were identified using the LASSO procedure and inserted into multivariate logistic regression models. We identified the different infiltration of immune cell types between the two groups. These results may provide new insights into the mechanism and pretreatment of AS.

## Methods

### Data download

We used the ‘GEOquery’ package of R software (4.0.2 version) to download the GSE100927 microarray data from the GEO database. The GSE100927 data set was based on the GPL17077 platform and contained 104 samples, including 69 atherosclerotic arteries and 35 healthy arteries.

### Differentially expressed genes (DEGs) identification

The raw data of GSE100927 was read by the affy package in R (version 3.6.1) and the rate monotonic algorithm was used for the background correction and data normalization. Hierarchical clustering analysis was used to group into 2 similar expression patterns of AS arteries and control. The principal component analysis (PCA) was performed to define probe quality control by the “stats” R package. DEGs were screened by the “limma” package. The cut-off criterion for statistically significant DEGs was determined as |log2FC|> 1 and P value < 0.05. The significant DEGs volcano map was drawn using the “ggplot2” package. The 15 top up-regulated and down-regulated genes were drawn using the “heatmap” package.

### Functional enrichment GO and KEGG analysis

Candidate genes enriched functions and pathways were identified by using the online DAVID tool (https://david.ncifcrf.gov/) [10], which was a free online tool that performed gene annotation and integrated discovery function. Gene ontology analysis (GO) is a common method for the annotation of gene products; it is also able to decide the defining biological characteristics of high-throughput genomic data. The Kyoto Encyclopedia of Genes and Genomes (KEGG) is a genetic function database that links functional information [11–13]. Both a P < 0.05 and a false discovery rate (FDR) < 0.05 (0.25) were considered to indicate statistical significance.

### PPI network and modular analysis

These significant DEGs were conducted in the STRING database (http://string-db.org) to evaluate the relationships of DEGs through protein–protein interaction (PPI) information [14]. The information on protein interaction relationship was imported into the Cytoscape software [15], which calculated the relationship between DEG encoding proteins in AS. Node degree was analyzed by the Network Analyzer plug-in, which determined the number of interconnections of PPI hub genes. Molecular complex detection (MCODE) was used to select PPI network modules on Cytoscape. The MCODE modules were more than 10 scores which were selected candidates. The nodules of module 1 were conducted for pathway enrichment analyses. Both a P < 0.05 and a false discovery rate (FDR) < 0.05 (0.25) were considered to indicate statistical significance.

### Enrichment analysis of gene ontology and KEGG by gene set enrichment analysis (GSEA).

The gene sequences were downloaded from the GSE100927 dataset. GSEA was used to analyze all gene sequences of the samples from two groups [16], and all genes were sequenced to indicate the trend of gene expression level between the two groups. We conducted GESA analysis and sorted genes according to the algorithm after importing gene annotation files and reference function sets, and then we get a gene sequence table. The GESA software analyzed all gene positions and accumulated them to get enrichment scores.

### Screening and verification of diagnostic markers

We used Least Absolute Shrinkage and Selection Operator (LASSO) logistic regression to perform feature selection to screen diagnostic markers for AS arteries [17]. The expression matrix data of GSE100927 were validated as independent matrix data and the diagnostic efficiency was verified as diagnostic markers. The LASSO algorithm was analyzed with the “glmnet” package. The LASSO Cox regression selected the AS features in the model. The LASSO cox regression model was based on the optimal lambda value in the RPI score for each patient. We found the 13 marker genes by using the LASSO cox regression model by the minimum criteria. We combined the genes from LASSO algorithms for further analysis. *P* < 0.05 was thought to be statistically significant.

### The abundance of immune cells in two groups

We analyzed the fractions of immune cells in each sample by CIBERSORT [18]. We performed a Wilcoxon rank-sum test to analyze these immune cells in the two groups. The differential densities of immune cells were shown and the differential colors were represented from low to high infiltrating levels.

### Statistical analysis

Data were expressed as means ± SD. Student’s paired t-test analyses were performed in two groups. The significant differences were considered when P < 0.05. R version 4.0.2 was applied to perform the analysis.

## Results

### DEG identification

**Fig. 1:**
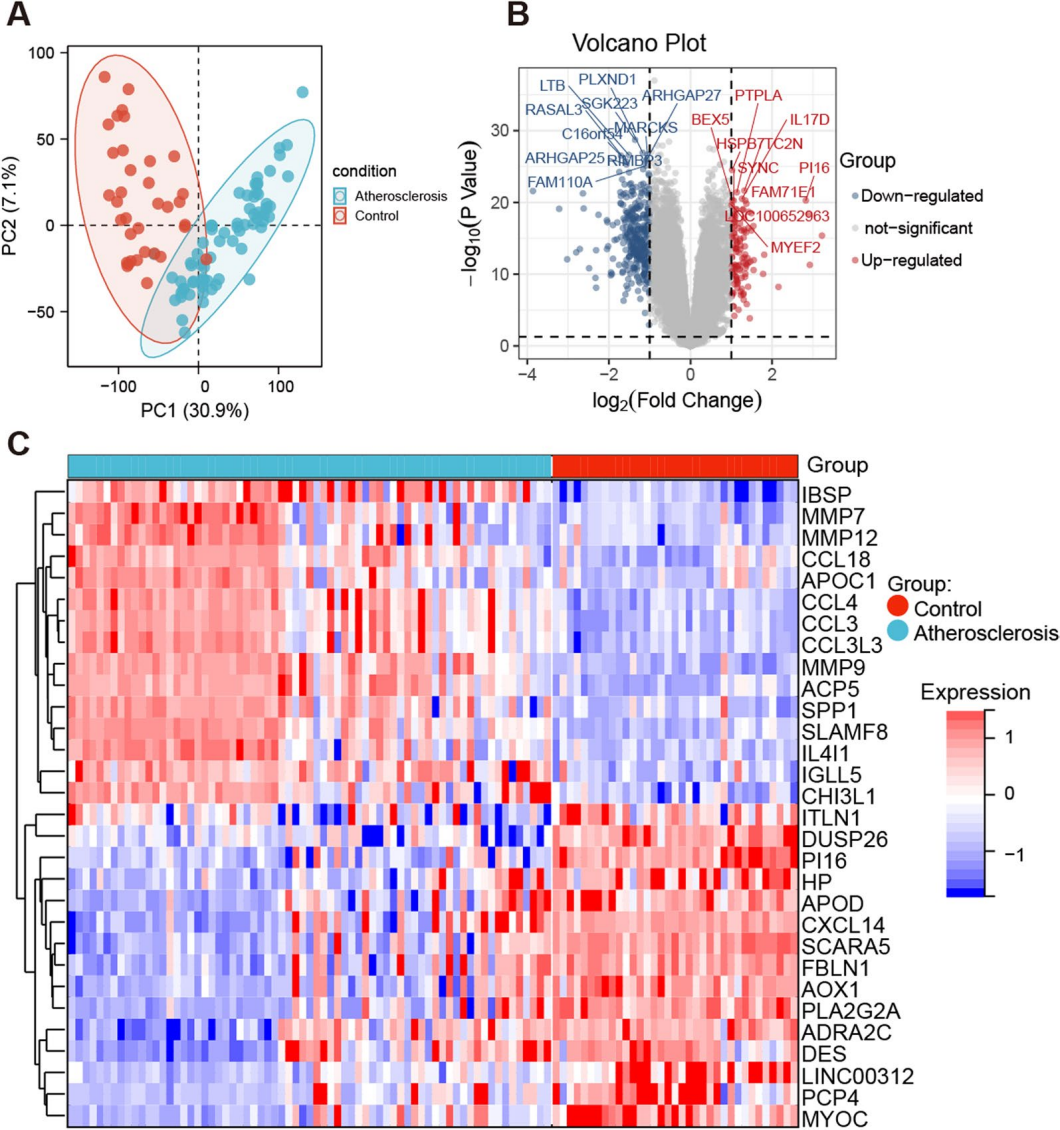
The DEGs identification of the GSE100927 dataset. **A** Principal component analysis (PCA) plot. **B** The volcano map of DEGs. When the threshold cutoff points were |logFC|≥ 1 and P < 0.05, 323 up-regulated genes (red) and 120 down-regulated genes (blue) were shown. The top 10 gene names were labeled. **C** The heatmap of the top 15 up-regulated genes and the top 15 down-regulated genes were presented

DEGs were separately identified to analyze the microarray data of GSE100927 from each chip. Figure 1A showed the PCA analysis between AS group and the control group. using|logFC|≥ 1 and P < 0.05 as the threshold cutoff points, 443 genes were highlighted. Among these, 323 genes were up-regulated and 120 genes were down-regulated in the AS group compared with the control group. The volcano diagram of 443 DEGs is presented in Fig. 1B. Figure 1C showed a heatmap of the top 15 up-regulated and top 15 down-regulated genes.

### GO term enrichment analyses and KEGG pathway analyses

**Fig. 2:**
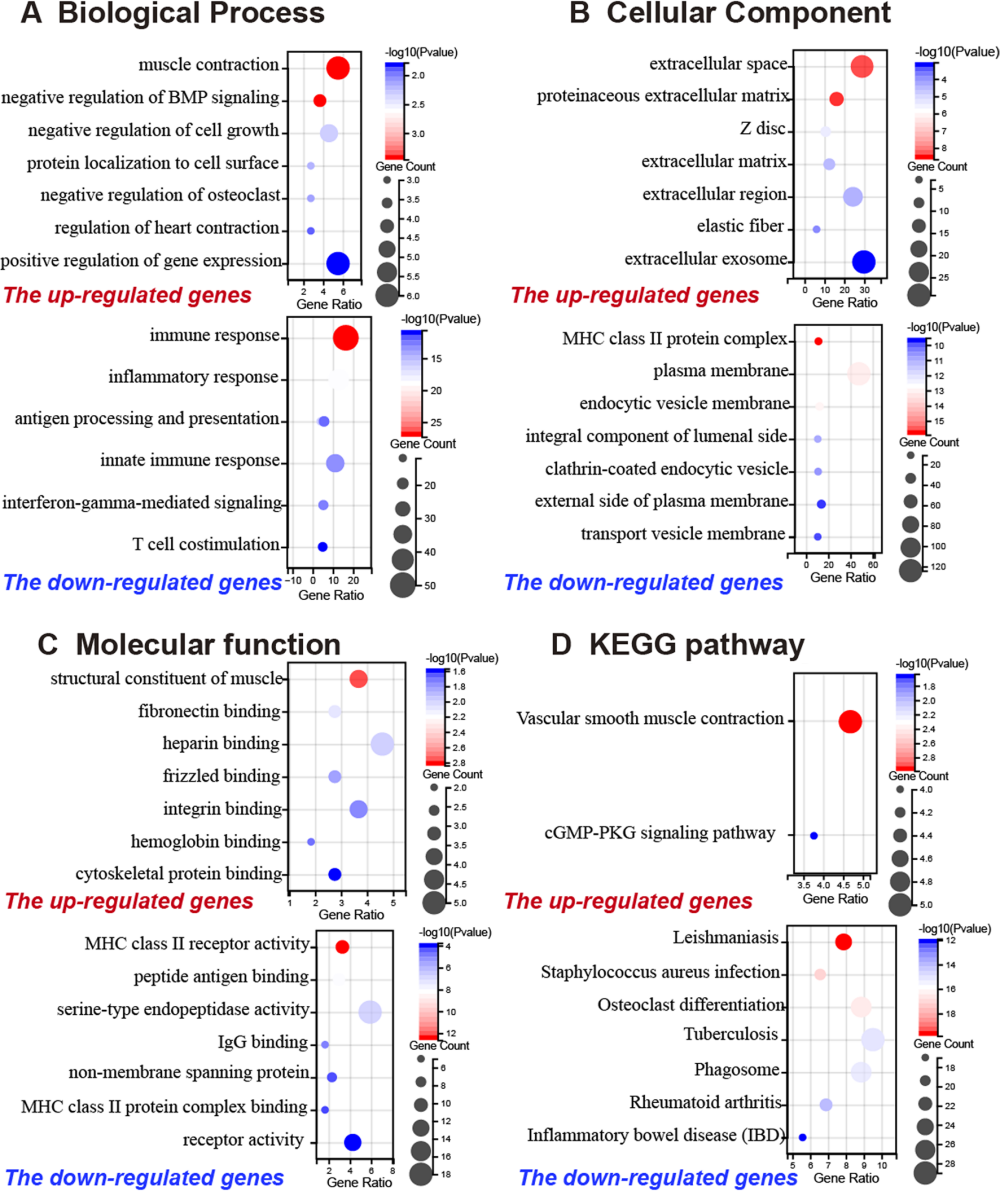
the significantly enriched GO and KEGG pathway in GSE100927. **A** BP analysis showed the top 7 terms in the up-regulated genes (upper) and the down-regulated genes(bottom), **B** CC analysis showed the top 7 terms in the up-regulated genes(upper) and the down-regulated genes(bottom). **C** MF analysis showed the top 7 terms in the up-regulated genes(upper) and the down-regulated genes(bottom). **D** The KEGG pathway analysis showed two terms in the up-regulated genes(upper) and showed the top 7 terms in the down-regulated genes(bottom)

GO and KEGG pathway enrichment analysis of DEGs were performed by using the DAVID online databases. DEGs were classified into biological processes (BP), cellular components (CC), and molecular functions (MF). The top 7 GO enrichment analysis results of DEGs were listed in Fig. 2. The up-regulated genes were mainly enriched in muscle contraction, negative regulation of BMP signaling pathway, and negative regulation of cell growth In BP, while the down-regulated DEGs were significantly enriched in BP such as immune response, inflammatory response, and innate immune response (Fig. 2A). The CC analysis demonstrated that the up-regulated genes were significantly enriched in proteinaceous extracellular matrix, extracellular space, extracellular matrix and region, while the down-regulated genes of DEGs were significantly enriched in MHC class II protein complex, plasma membrane, and endocytic vesicle membrane (Fig. 2B). In addition, MF analysis showed that the up-regulated genes in DEGs were enriched in structural constituent of muscle, fibronectin binding, and heparin binding, while the down-regulated genes were enriched in MHC class II receptor activity, peptide antigen binding, and serine-type endopeptidase activity (Fig. 2C).

Figure 2D showed the significant enrichment pathways of the up-regulated DEGs and down-regulated DEGs analyzed by KEGG analysis. The up-regulated DEGs were significantly enriched in vascular smooth muscle contraction and cGMP-PKG signaling pathway, while the down-regulated DEGs were enriched in Leishmaniasis, Tuberculosis, Phagosome, and Osteoclast differentiation.

### Identification of hub genes and modular screening

**Fig. 3:**
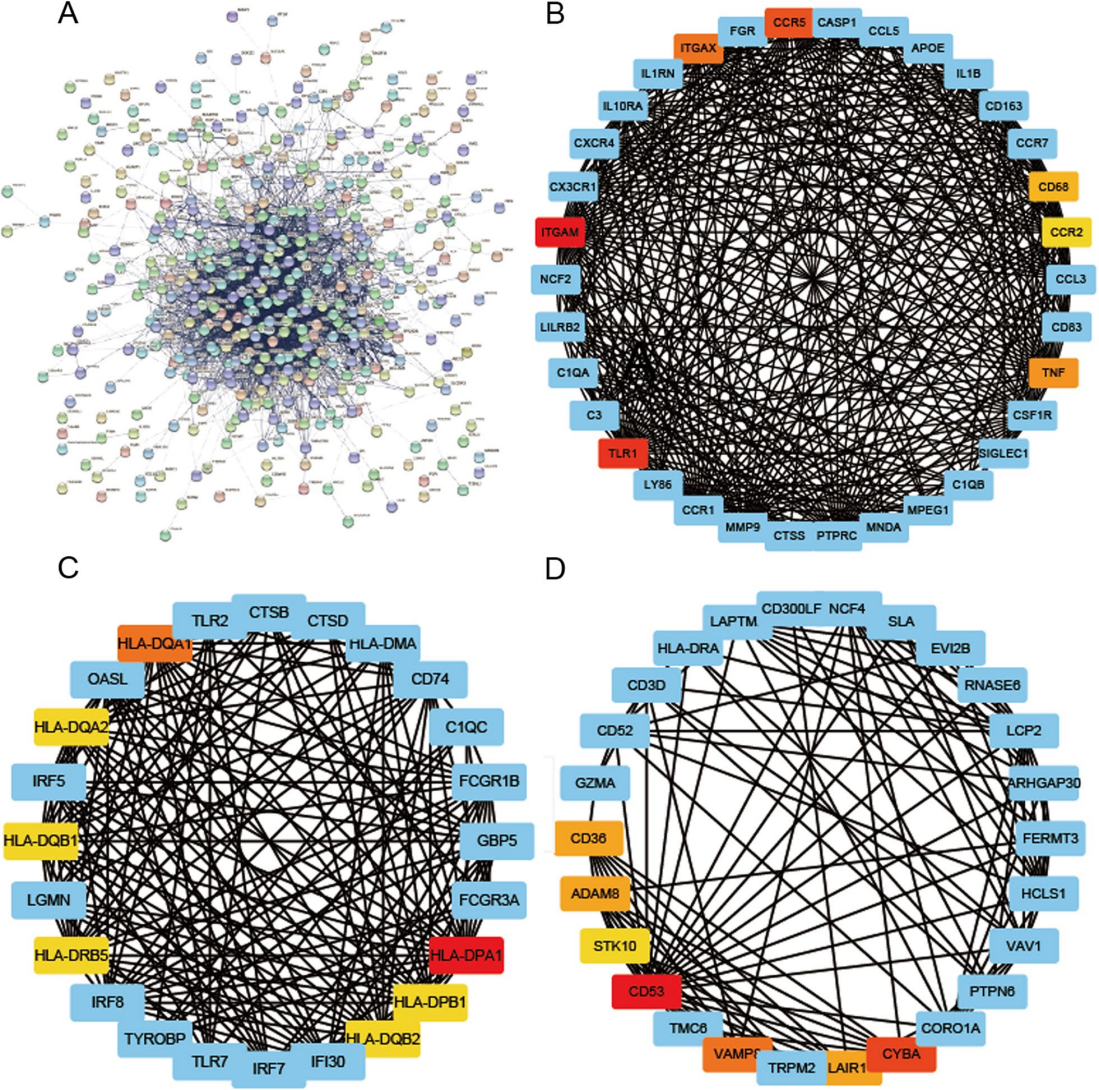
PPI networks of DEGs and key modules. **A** 421 DEGs were filtered into the STRING online database; **B** Module 1 comprises 34 nodes and 111 edges; **C** Module 2 comprises 41 nodes and 15 edges; **D** Module 3 comprises 40 nodes and 18 edges; the top 3 modules showed the top 7 important genes from each module

We employed the STRING online database to construct a protein–protein interaction (PPI) network of DEGs. DEGs were imported into the STRING database and a total of 421 nodes and 3529 edges were obtained with a scoring value > 0.4 (Fig. 3A). The cytoHubba plugin of Cytoscape software was used to identify hub genes in the PPI network. The top 10 hub genes with higher degrees of connectivity were PTPRC, TYROBP, TNF, ITGAM, SPI1, LCP2, CSF1R, TLR2, CCR5, and ITGAX. The higher degree and closeness of the top 10 hub genes were listed in Table 1.

**Table 1:** 10 hub genes identified by Cytohubba

Hub gene	Degree	Closeness	Betweenness	Stress	Clustering Coefficient	logFC	P Value
PTPRC	121	229	9277	126,404	0.250	− 1.228	6.93E−17
TYROBP	112	222	6619	96,570	0.257	− 1.572	2.61E−17
*TNF*	106	223	17,237	178,086	0.221	− 1.458	2.31E−23
ITGAM	101	217	4327	72,320	0.300	− 1.483	1.20E−16
SPI1	97	214	5750	74,026	0.290	− 1.536	2.61E−19
LCP2	90	209	3011	53,996	0.301	− 1.326	9.36E−19
CSF1R	81	206	4427	70,286	0.339	− 1.614	3.17E−23
CCR5	77	204	3087	59,934	0.370	− 1.652	2.28E−20
TLR2	77	204	1898	44,128	0.381	− 1.035	8.47E−11
ITGAX	76	203	1619	31,974	0.356	− 1.327	3.06E−12

Furthermore, the MCODE plugin of Cytoscape was used to identify gene cluster modules and 6 significant gene cluster modules were obtained. The top 3 key modules from the PPI network were selected: Module 1(significant gene cluster) contained 34 nodes and 361 edges with a score of 21.87. The top 7 genes were ITGAM, TLR1, CCR5, ITGAX, TNF, CD68, and CCR2 (Fig. 3B). Module 2 comprised 41 nodes and 288 edges with a score of 14.40. The top 7 genes were HLA-DPA1, HLA-DQA1, HLA-DBP1, HLA-DQB1, HLA-DRB5, HLA-DQB2, and HLA-DQA2 (Fig. 3C). Module 3 consisted of 40 nodes and 180 edges with a score of 9.23. The top 7 genes were CD53, CYBA, VAMP8, ADAM8, LAIR1, CD36, and STK10 (Fig. 3D).

### Identification of KEGG pathway analyses of the top 3 Modules

**Fig. 4:**
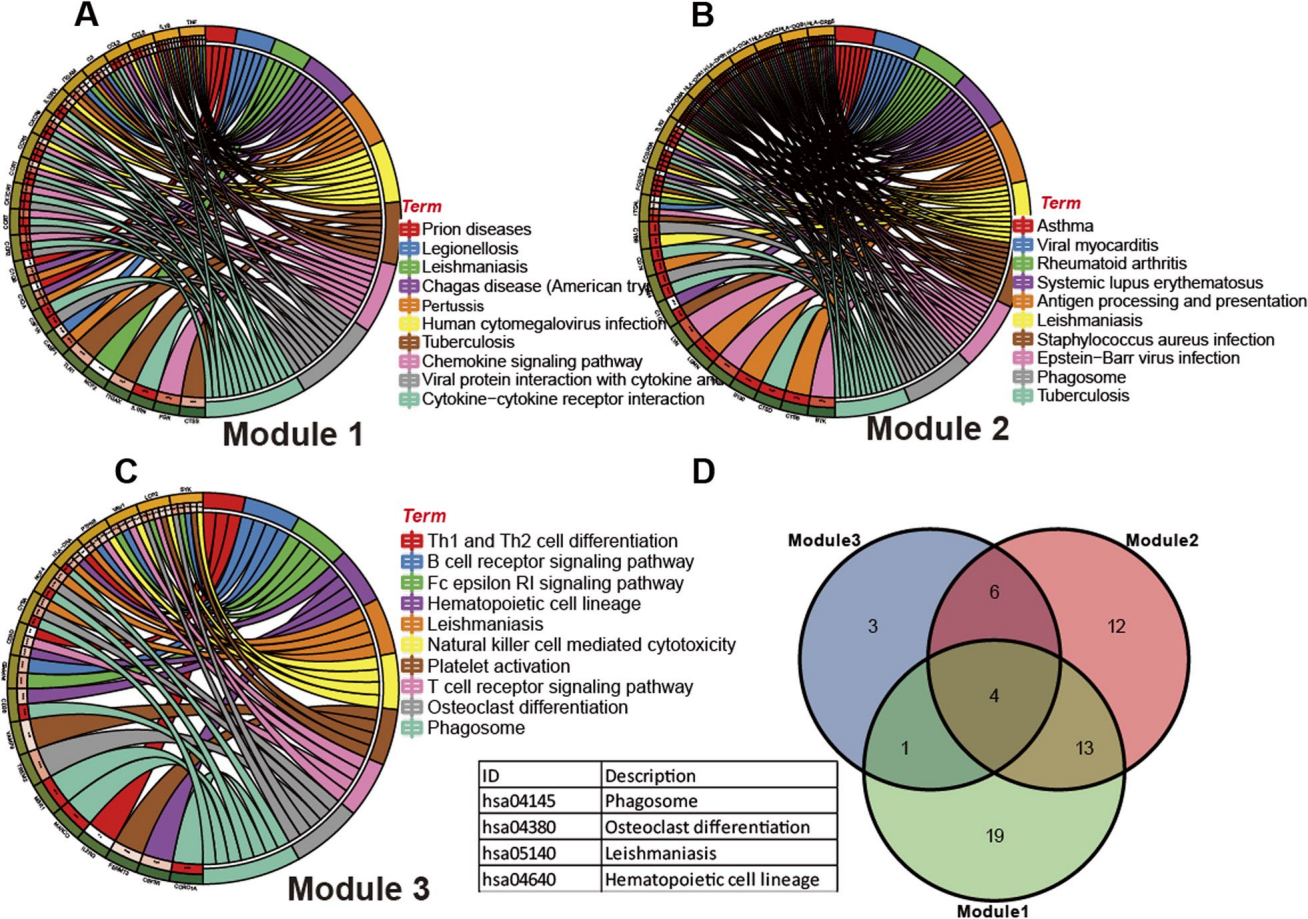
The KEGG analyses of the top 3 modules. **A** KEGG pathway enrichment analysis of module 1 was associated with 37 KEGG pathways and showed the top 10 pathways. **B** Module 2 was associated with 35 KEGG pathways and showed the top 10 pathways. **C** Module 3 was associated with 14 KEGG pathways and showed the top 10 pathways. **D** The 4 common KEGG pathways of the top 3 modules were shown by Venn diagram

The top 3 modules with high scores indicated that they may play an important role in the PPI network. KEGG pathway terms of these genes for the top 3 modules were analyzed using DAVID online software. The enrichment analysis results showed that Module 1 enriched 37 KEGG pathways (Additional file 1: Table S1), Module 2 enriched 35 KEGG pathways (Additional file 1: Table S2), and Module 3 enriched 14 KEGG pathways (Additional file 1: Table S3). Module 1 was mainly enriched in cytokine-cytokine receptor interaction, Pertussis, Chemokine signaling pathway, and Tuberculosis (Fig. 4A). Module 2 was mainly enriched in Staphylococcus aureus infection, Antigen processing and presentation, Tuberculosis, and phagosome (Fig. 4B). Module 3 was mainly enriched in phagosome and osteoclast differentiation, respectively (Fig. 4C). Furthermore, we found four common KEGG pathways that were Leishmaniasis, phagosome, Hematopoietic cell lineage, and Osteoclast differentiation in the top 3 modules (Fig. 4D).

### Function and enrichment analysis of GSEA.

**Fig. 5:**
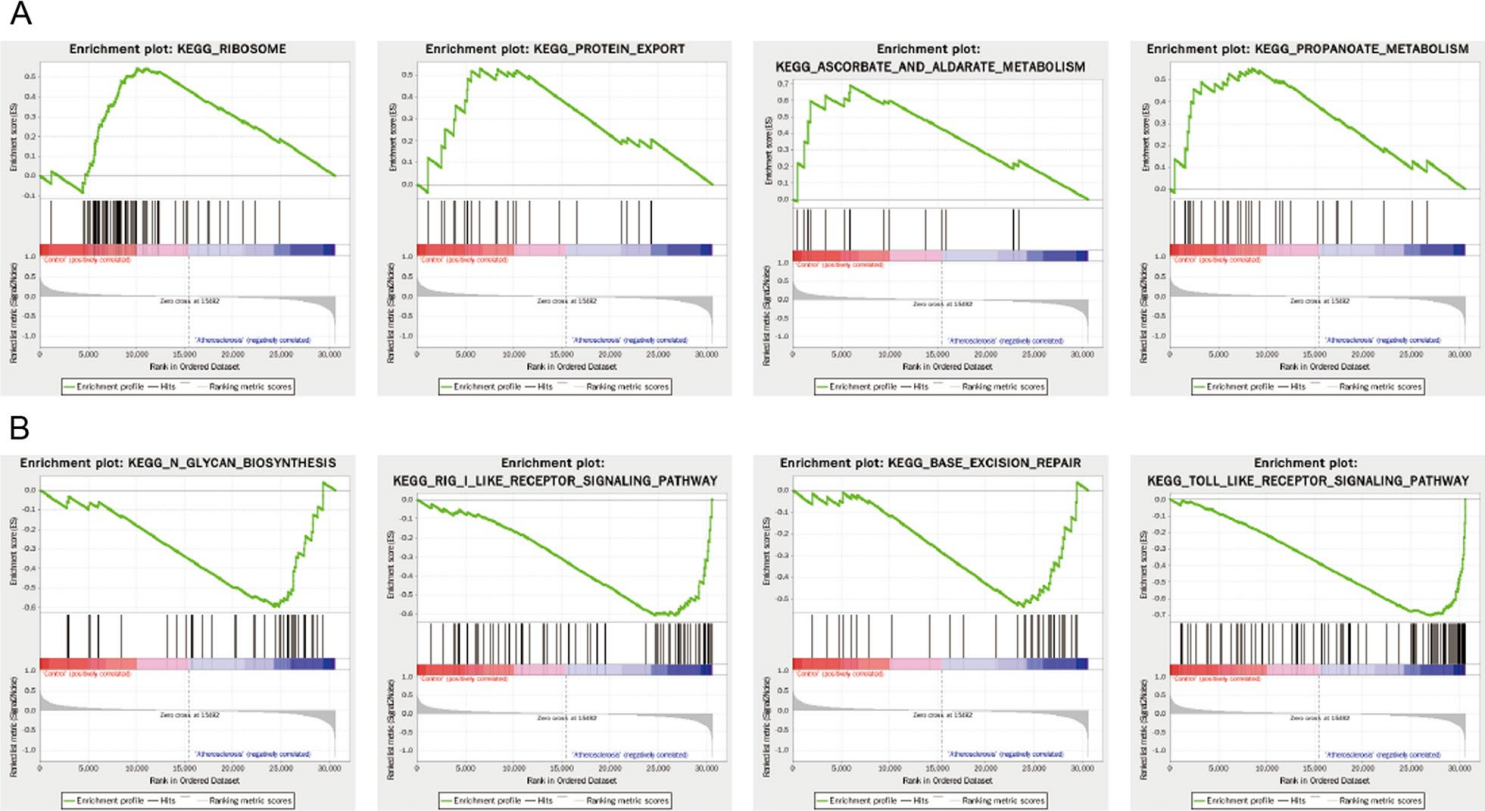
Eight significant enrichment plots of functional enrichment analysis of DEGs between AS and control group in GSE100927 by GSEA. **A** The enrichment plots in the up-regulated gene sets: ribosome, protein export, ascorbated and aldarate metabolism, and propanoate metabolism. **B** The enrichment plots in the down-regulated gene sets: N-glycanbiosynthesis, rig-I-like receptor signaling pathway, base excision repair, and toll-like receptor signaling pathway

The DEG expression profile of GSE100927 was uploaded into Gene Set Enrichment Analysis (GSEA) software. GSEA analysis showed that 122/186 gene sets were up-regulated, while 64/186 gene sets were down-regulated between AS group and the control group. Table 2 ranks the most significant enrichments in the up-regulated gene sets (NES > 0.8 and P value < 0.05) and down-regulated gene sets (NES < − 1.3 and P value < 0.05) based on normalized enrichment score (NES). We presented the top 4 most significant plots in the up-regulated (Fig. 5A) and in the down-regulated gene set (Fig. 5B). The up-regulated gene sets were significantly enriched for ribosome, ascorbated and aldarate metabolism, propanoate metabolism, and cardiac muscle contraction, while the down-regulated gene sets were significantly enriched for N-glycanbiosynthesis, rig-I-like receptor signaling pathway, base excision repair, and toll-like receptor signaling pathway, receptively.

**Table 2:** KEGG pathway enrichment analysis of DEGs in AS group and control group using GSEA. ES: Enrichment Score; NES: Normalized Enrichment Score

Gene set name	ES	NES	P value	Count
*The GESA analysis for control*				
KEGG_RIBOSOME	0.545	1.759	0.024	80
KEGG_ASCORBATE_AND_ALDARATE_METABOLISM	0.691	1.579	0.021	15
KEGG_PROPANOATE_METABOLISM	0.552	1.564	0.017	29
KEGG_CARDIAC_MUSCLE_CONTRACTION	0.504	1.549	0.021	68
KEGG_TIGHT_JUNCTION	0.423	1.445	0.046	126
*The GESA analysis for AS*				
KEGG_N_GLYCAN_BIOSYNTHESIS	− 0.595	− 1.782	0.006	43
KEGG_RIG_I_LIKE_RECEPTOR_SIGNALING_PATHWAY	− 0.608	− 1.769	0.002	65
KEGG_BASE_EXCISION_REPAIR	− 0.535	− 1.697	0.012	32
KEGG_TOLL_LIKE_RECEPTOR_SIGNALING_PATHWAY	− 0.706	− 1.63	0.002	94
KEGG_O_GLYCAN_BIOSYNTHESIS	− 0.7	− 1.629	0.004	24
KEGG_NON_SMALL_CELL_LUNG_CANCER	− 0.5	− 1.627	0.004	53
KEGG_NATURAL_KILLER_CELL_MEDIATED_CYTOTOXICITY	− 0.691	− 1.619	0.006	118
KEGG_T_CELL_RECEPTOR_SIGNALING_PATHWAY	− 0.663	− 1.604	0.008	104
KEGG_APOPTOSIS	− 0.578	− 1.602	0.025	85
KEGG_ENDOCYTOSIS	− 0.445	− 1.585	0.006	166
KEGG_ACUTE_MYELOID_LEUKEMIA	− 0.537	− 1.579	0.012	56
KEGG_GLYCOSAMINOGLYCAN_BIOSYNTHESIS_HEPARAN_SULFATE	− 0.621	− 1.571	0.006	25
KEGG_ANTIGEN_PROCESSING_AND_PRESENTATION	− 0.728	− 1.568	0.01	77
KEGG_EPITHELIAL_CELL_SIGNALING_IN_HELICOBACTER_PYLORI_INFECTION	− 0.627	− 1.568	0.021	65
KEGG_B_CELL_RECEPTOR_SIGNALING_PATHWAY	− 0.703	− 1.567	0.008	71
KEGG_FC_GAMMA_R_MEDIATED_PHAGOCYTOSIS	− 0.61	− 1.565	0.006	88
KEGG_SULFUR_METABOLISM	− 0.78	− 1.564	0.002	11
KEGG_NOTCH_SIGNALING_PATHWAY	− 0.529	− 1.558	0.029	45
KEGG_VIBRIO_CHOLERAE_INFECTION	− 0.581	− 1.557	0.028	52
KEGG_PEROXISOME	− 0.415	− 1.556	0.034	74
KEGG_ALZHEIMERS_DISEASE	− 0.382	− 1.555	0.013	139
KEGG_CYTOSOLIC_DNA_SENSING_PATHWAY	− 0.669	− 1.55	0.022	48
KEGG_GLYCOSPHINGOLIPID_BIOSYNTHESIS_LACTO_AND_NEOLACTO_SERIES	− 0.556	− 1.549	0.019	25
KEGG_LEUKOCYTE_TRANSENDOTHELIAL_MIGRATION	− 0.583	− 1.548	0.017	112
KEGG_THYROID_CANCER	− 0.524	− 1.544	0.025	29
KEGG_AUTOIMMUNE_THYROID_DISEASE	− 0.785	− 1.533	0.006	47
KEGG_GLYCOSPHINGOLIPID_BIOSYNTHESIS_GLOBO_SERIES	− 0.759	− 1.533	0.021	13
KEGG_PANTOTHENATE_AND_COA_BIOSYNTHESIS	− 0.733	− 1.529	0.008	15
KEGG_LYSOSOME	− 0.791	− 1.509	0.004	112
KEGG_CHEMOKINE_SIGNALING_PATHWAY	− 0.638	− 1.5	0.025	172
KEGG_GLYCOSPHINGOLIPID_BIOSYNTHESIS_GANGLIO_SERIES	− 0.577	− 1.419	0.044	14
KEGG_PRIMARY_IMMUNODEFICIENCY	− 0.822	− 1.415	0.029	35
KEGG_PHOSPHATIDYLINOSITOL_SIGNALING_SYSTEM	− 0.376	− 1.365	0.031	76

### Screening and verification of diagnostic markers

**Fig. 6:**
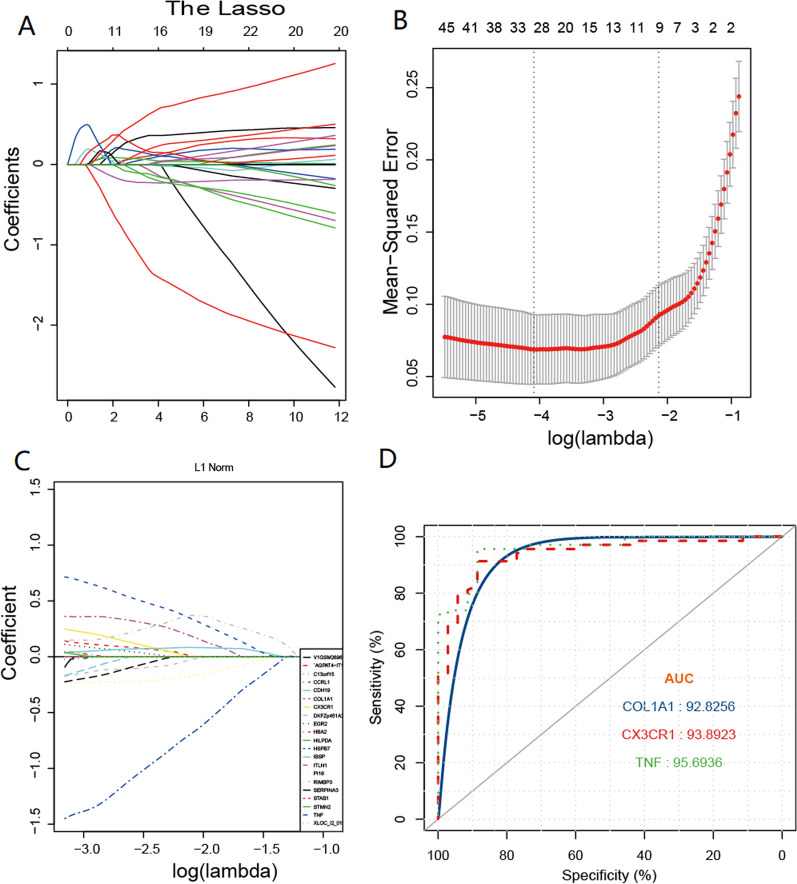
LASSO Cox regression analysis and ROC curve. **A** LASSO coefficient profiles of GSE100927. A coefficient profile plot was produced against the log (λ) sequence. **B** Selection of tuning parameter (λ) in the LASSO model in the minimum criteria. Dotted vertical lines were shown at the optimal values using the minimum criteria. A λ value of 0.1013 with log (λ) of − 2.290 was chosen. **C** Seventeen survival-related genes were selected by LASSO Cox regression analysis. **D** ROC analysis showed the diagnostic value of risk score and ROC curves presenting the discrimination ability of TNF, CX3CR1, and COL1A1. The AUC curve of the ROC curve was 0.96, 0.94, and 0.93, indicating better predictive power

To obtain the diagnostic markers genes, Least Absolute Shrinkage and Selection Operator (LASSO) Cox regression analysis was performed based on the gene expression of DEGs. We used the LASSO logistic regression algorithm to screen the diagnostic markers from 443 DEGs. We randomly selected 70% of samples in GSE10927 as the training set construction of the models and the remaining 30% of samples as the test set (internal validation set). A coefficient profile plot was produced against the log (lambda) sequence. Selection of tuning parameter (lambda) in the LASSO model used tenfold cross-validation via minimum criteria. Figure 6A,B show the procedure of screening. We identified the 17 hub genes (Table 3). The 17 hub genes were performed by the multivariate Cox regression analysis to calculate the coefficient of signature, which represented the weight of each gene (Fig. 6C). In order to validate the robustness of the top 3 genes signature, ROC analysis was performed for the validation cohort. The area under the curve (AUC) for TNF, CX3CR1and COL1R1 was 0.96, 0.94, and 0.93, respectively (Fig. 6D). These results indicated that the top 3-genes prognostic model has a high predictive ability of AS.

**Table 3:** the regression coefficients of LASSO Cox regression analysis

GENE NAMES	COEF	EXPCOEF
COL1A1	0.8425	2.3221
TNF	0.5788	1.7839
CX3CR1	0.3778	1.4591
STAB1	0.3552	1.4265
IBSP	0.1878	1.2065
FOSB	0.0911	1.0954
DKFZp451A211	− 0.0667	0.9354
ABCA8	− 0.2773	0.7578
PI16	− 0.2934	0.7457
SERPINA3	− 0.3621	0.6962
TC2N	− 0.3869	0.6791
DUSP26	− 0.4224	0.6555
CCRL1	− 0.5781	0.561
CDH19	− 0.6179	0.5391
C13orf33	− 1.0437	0.3522
XLOC_l2_013193	− 1.0651	0.3447
HSPB7	− 1.5339	0.2157

### Differential abundance of infiltrating immune cells in AS

**Fig. 7:**
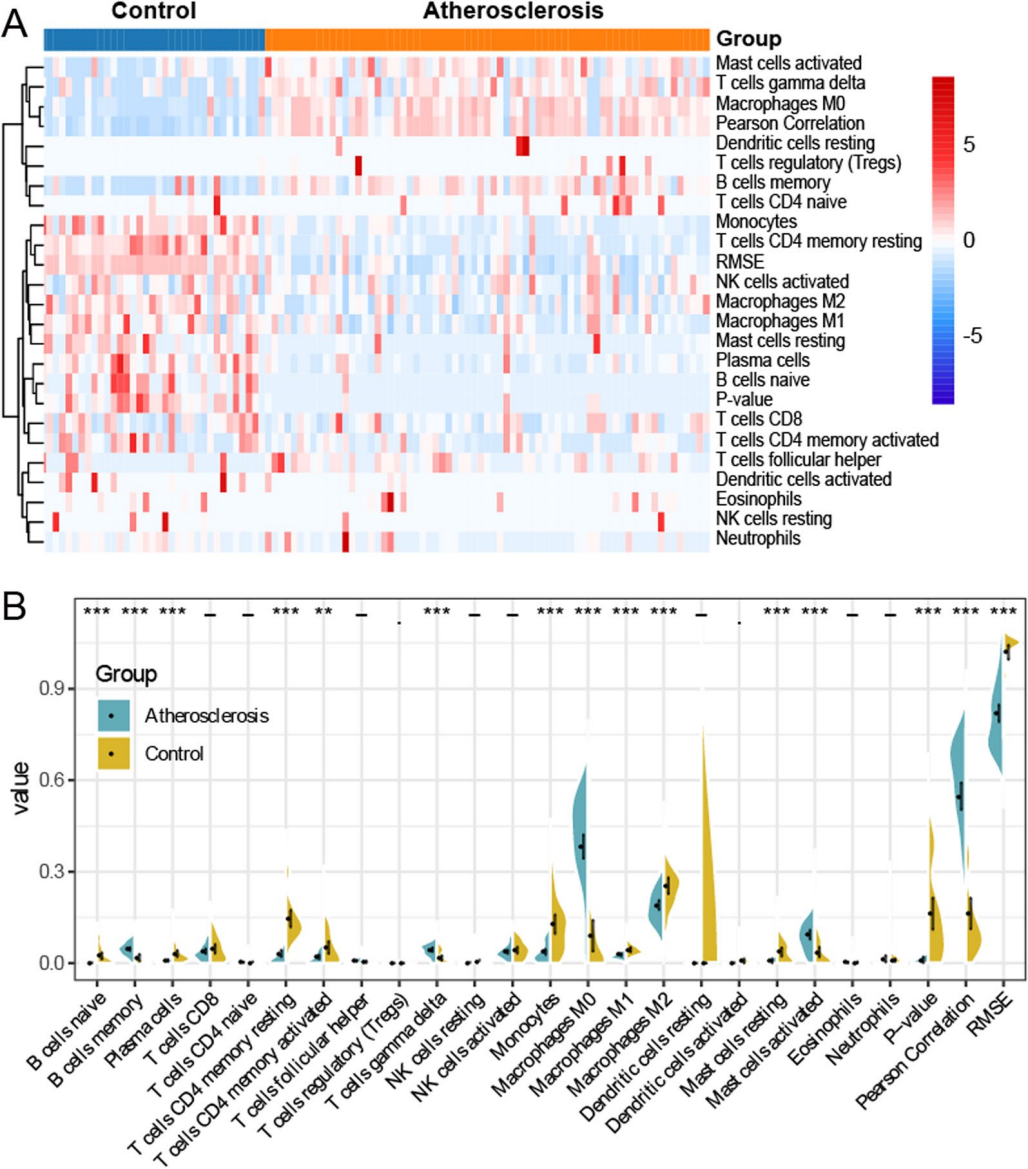
Distribution of immune cells in AS and control groups. **A** The heatmap showed the differential infiltration of immune cells in AS and control groups. **B** Wilcoxon rank-sum test compared the differential proportions of immune cells in AS and control groups. **P < 0.01; ***P < 0.001, compared to control group

The KEGG pathway enrichment analyses were related to infection, such as Leishmaniasis, and phagosome. To assess the potential associations, we analyzed the different immune cell types between AS group and the control group. The heatmap presented the different immune cell infiltration patterns between the two groups, where the colors displayed the infiltration density (Fig. 7A). In Addition, we utilized the Wilcoxon rank-sum test to compare the difference and discovered these immune cells had a significantly higher infiltrating density in AS groups, including M0 macrophages, T cells gamma delta, memory B cells, as well as activated mast cells (Fig. 7B). On the other hand, we discovered that several immune cells conferred a significantly lower infiltrating density in AS groups, such as monocytes, T cells CD4 memory resting, M2 macrophages, and activated NK cells.

## Discussion

Atherosclerosis (AS), as a metabolic syndrome, posed a serious threat to human health and caused a series of complications affecting the cardiovascular system, including acute myocardial infarction and cerebral infarction [6]. It is well known that AS was initiated by lipid-mediated vascular inflammation, which enhanced continual monocyte recruitment and foam-cell formation in the arterial wall. Roy reported that AS is characterized by imbalanced lipid metabolism [5]. The GSE100927 data set successfully extracted 443 DEGs between AS and the control group. Functional annotations of DEGs are mainly assessed through the analysis of GO and KEGG pathway enrichment.

BP analyses demonstrated that the up-regulated DEGs were mainly enriched in muscle contraction and negative regulation of the BMP signaling pathway, while the down-regulated DEGs were enriched in immune response and inflammatory response [3]. These findings are in line with the hypothesis, which stated that AS development is a complicated inflammatory process with immune reactions and is associated with decreased vascular smooth muscle contraction and proliferation [5, 6]. In addition, the enriched KEGG pathways of up-regulated DEGs found osteoclast differentiation and phagosome. Previous studies have shown that lipid phagocytosis is a crucial process involved in atherogenesis [19, 20] and the inhibition of osteoclastic differentiation impaired calcium resorption in atherosclerotic plaques [21]. Our result was consistent with these findings that the up-regulated genes of DEGs were enriched in vascular smooth muscle contraction, while the down-regulated genes were enriched in Leishmaniasis, Phagosome, and Osteoclast differentiation.

We identified the top 10 hub genes based on degree value by using Cytoscape software, including PTPRC, TYROBP, TNF, ITGAM, SPI1, LCP2, CSF1R, CCR5, and ITGAX. Nie reported that PTPRC was considered to be an immune marker gene in atherosclerosis development [22]. Liu reported that the upregulated TYROBP was identified as a common hub gene and as an immune-responsive hub gene in the advanced atherosclerotic plaques [23]. Lu found that TYROBP expression was significantly higher in the low-grade glioma tissues compared to the normal tissues [24]. TNF-αis a pro-atherogenic stimulus on vascular endothelial cells. Yuan supported the evidence of the causal associations of increased TNF levels with a higher risk of cardiovascular diseases, including AS [25]. The TNF-α expression was higher in obese patients with cardiovascular disease (CAD) [26]. Wei reported that Csf1r inhibited macrophage proliferation and impaired the progression of AS [27]. Lin reported that CCL5/CCR5 axis activation promoted the proliferation and the phenotypic switching of vascular smooth muscle cells [28]. A study found CCR5 is related to better survival of triple-negative breast cancer through improvement tumor microenvironment [29]. Zhao reported that ITGAM is a potential diagnostic and prognostic biomarker of unstable atherosclerotic plaque-related stroke, which will be a novel therapeutic target and the development of novel management strategies [30]. Therefore, these hub genes are closely related to the pathological process of AS and may play a key role in the early diagnosis of AS patients. The PPI network construction highlighted these important genes, which revealed the characteristics of atherosclerotic lesions.

The functions of the genes in three important modules from PPI were selected to further perform the KEGG pathway enrichment analysis. The common three KEGG enrichment pathways were osteoclast differentiation, phagosome, and Leishmaniasis. These findings are in line with other research which reported that AS development is a result of imbalanced lipid metabolism and the AS progression was mediated by the phagosome of macrophages and smooth muscle cells [5]. The findings were consistent with our previous research which reported that the transdifferentiation of vascular smooth muscle cells mediated the AS process [9]. The GSEA results showed that these genes were enriched in ribosome, N-glycanbiosynthesis, and rig-I-like receptor signaling pathway, which were in line with the KEGG pathway analysis identified phagosome, Leishmaniasis, and osteoclast differentiation. Some hub genes, including TYROBP, TNF, SP11, LCP2 and CSF1R, played important roles in phagosome [31, 32]. TNF and SP11 are related to leishmaniasis infection [33, 34]. Moreover, CCR5 is strongly associated with Leishmaniasis in HIV-1-infected patients [35] and the CCR5 reduction accelerated osteoclastogenesis [36]. Some studies found that these hub genes, such as PTPRC, TNF, ITGAM, SP11, and ITGAX, are significantly associated with osteoclast differentiation [34, 37, 38]. CSF1R expression elucidated the impact of specific miRNA-mRNA interactions in osteoclastogenesis [39], All in all, these hub genes are highly connected to three enriched pathways.

To evaluate the prognostic genes based on DEGs of GSE100927, we applied the LASSO Cox regression model to construct a prognostic gene signature. We identified a gene signature (TNF, CX3CR1, and COL1R1) involved in AS. The AUC value of the three-gene signature suggested the prediction ability and the prognostic values of these gene signatures. TNF-α could induce the up-expression of CX3CR1 in endothelial cells and BV-2 microglial cells [40, 41]. Morimura reported that the peritoneal macrophages of the CX3CR1 (−/−) mice expressed lower expression levels of TNF-α, IL-1β, and IL-6 [42]. Garre suggested the highlight CX3CR1^high^ monocytes and TNF-α as potential therapeutic targets for preventing infection-induced cognitive dysfunction [43]. The novel insight provides new noninvasive methods and may inform early prediction and early intervention strategies for AS patients.

AS is a chronic inflammatory disease involving multiple types of immune cells. Chistiakov reported that Tregs mediated the immune response and the secretion of anti-inflammatory cytokines IL-10 and TNF-beta [44]. CD8 + T cells enhanced the secretion of IFN-γ, IL-2, and TNF-α [45]. DC promoted the activating of the TNF-α/NF-κB/CXCR-4 pathway [46]. Ni reported that CX3CR1 deficiency caused a significant increase in inflammatory monocyte/macrophage infiltration [47]. Collagen type I(COL1) is remodeled in atherosclerotic plaque and the COL1 degradation is associated with mortality in patients with AS [48]. These results were consistent with the immune cell infiltration in AS groups.

The main pathological process of AS involved vascular endothelial cell damage, lipid deposition, and plaque formation [2]. Our results showed that the down-regulated genes enriched in Leishmaniasis, Phagosome, and Osteoclast differentiation. We identified the top 10 hub genes. Some hub genes(TYROBP, TNF, SP11, LCP2, and CSF1R) played a key role in phagosome. TNF, SP11, and CCR5 are related to leishmaniasis infection. Other hub genes(PTPRC, TNF, ITGAM, SP11, and ITGAX) are significantly associated with osteoclast differentiation. We constructed a gene signature (TNF, CX3CR1, and COL1R1). The AUC value suggested the prediction ability and the prognostic value. Three genes were associated with immune cell infiltration in AS patients. Our findings explored the regulatory network of AS and represented a novel diagnostic and therapeutic target for AS.

There are also some limitations in our study. Firstly, the hub genes and the gene signature were analyzed using gene expression profiles of human samples from online public databases; further studies using more human samples will be required to validate these marker genes by WB experiments. Secondly, more experiments need to examine these potential mechanisms related to the phagosome, Leishmaniasis, and osteoclast differentiation in tissue samples from patients with AS.

In summary, our study displays a new comprehensive bioinformatics analysis of DEGs that might be involved in the AS progress. We identified 10 hub genes and three modules that were highly associated with AS. The KEGG pathways were mainly enriched in phagosome, Leishmaniasis, and Osteoclast differentiation. We developed and validated a three-gene signature to predict the prognosis of AS. These novel molecular targets are principal candidate genes for further investigations into biomarkers and molecular mechanisms.

## Supplementary Information

**Additional file 1. Table S1.** The KEGG pathway of Module 1, Related to Figure 4A. **Table S2.** The KEGG pathway of Module 2, Related to Figure 4B. **Table S3.** The KEGG pathway of Module 3, Related to Figure 4C.

## Data Availability

The datasets analyzed during the current study are available in the GEO repository, (https://www.ncbi.nlm.nih.gov/geo, accession number GSE100927)..

## Abbreviations

AS: Atherosclerosis
GEO: Gene Expression Omnibus
GO: Gene ontology
BP: Biological processes
CC: Cellular components
MF: Molecular functions
KEGG: Kyoto Encyclopedia of Genes and Genomes
DEGs: Differentially expressed genes
LASSO: Least absolute shrinkage and selection operator
VSMC: Vascular smooth muscle cell
LDL: Low-density lipoprotein
mtDNA: Mitochondrial DNA
PPI: Protein–protein interaction
PCA: Principal component analysis
FDR: False discovery rate
MCODE: Molecular complex detection
ROC: Receiver-operator characteristic
STRING: Search Tool for the Retrieval of Interacting Genes/Proteins
GSEA: Gene set enrichment analysis
MHC: Major histocompatibility complex
TNF-α: Tumor necrosis factor-α
CX3CR1: CX3C receptor 1

## References

[1] Lanzer P, Hannan F, Lanzer J, Janzen J, Raggi P, Furniss D (2021). Medial arterial calcification: JACC state-of-the-art review. J Am Coll Cardiol.

[2] Xu S, Ilyas I, Little P, Li H, Kamato D, Zheng X (2021). Endothelial dysfunction in atherosclerotic cardiovascular diseases and beyond: from mechanism to pharmacotherapies. Pharmacol Rev.

[3] Durham A, Speer M, Scatena M, Giachelli C, Shanahan C (2018). Role of smooth muscle cells in vascular calcification: implications in atherosclerosis and arterial stiffness. Cardiovasc Res.

[4] Wesseling M, Sakkers T, de Jager S, Pasterkamp G, Goumans M (2018). The morphological and molecular mechanisms of epithelial/endothelial-to-mesenchymal transition and its involvement in atherosclerosis. Vascul Pharmacol.

[5] Roy P, Orecchioni M, Ley K (2021). How the immune system shapes atherosclerosis: roles of innate and adaptive immunity. Nat Rev Immunol.

[6] Libby P (2021). The changing landscape of atherosclerosis. Nature.

[7] Mezentsev A, Bezsonov E, Kashirskikh D, Baig MS, Eid AH, Orekhov A (2021). Proatherogenic sialidases and desialylated lipoproteins: 35 years of research and current state from bench to bedside. Biomedicines.

[8] Bezsonov EE, Sobenin IA, Orekhov AN (2021). Immunopathology of atherosclerosis and related diseases: focus on molecular biology. Int J Mol Sci.

[9] Chen J, Zhang X, Millican R, Sherwood J, Martin S, Jo H (2021). Recent advances in nanomaterials for therapy and diagnosis for atherosclerosis. Adv Drug Deliv Rev.

[10] da Huang W, Sherman BT, Lempicki RA (2009). Systematic and integrative analysis of large gene lists using DAVID bioinformatics resources. Nat Protoc.

[11] Kanehisa M, Goto S (2000). KEGG: kyoto encyclopedia of genes and genomes. Nucleic Acids Res.

[12] Kanehisa M, Sato Y, Kawashima M (2022). KEGG mapping tools for uncovering hidden features in biological data. Protein Sci.

[13] Kanehisa M, Furumichi M, Sato Y, Ishiguro-Watanabe M, Tanabe M (2021). KEGG: integrating viruses and cellular organisms. Nucleic Acids Res.

[14] Szklarczyk D, Gable AL, Nastou KC, Lyon D, Kirsch R, Pyysalo S (2021). The STRING database in 2021: customizable protein-protein networks, and functional characterization of user-uploaded gene/measurement sets. Nucleic Acids Res.

[15] Shannon P, Markiel A, Ozier O, Baliga NS, Wang JT, Ramage D (2003). Cytoscape: a software environment for integrated models of biomolecular interaction networks. Genome Res.

[16] Subramanian A, Tamayo P, Mootha V, Mukherjee S, Ebert B, Gillette M (2005). Gene set enrichment analysis: a knowledge-based approach for interpreting genome-wide expression profiles. Proc Natl Acad Sci.

[17] Efendi A, Ramadhan HW, editors. Parameter estimation of multinomial logistic regression model using least absolute shrinkage and selection operator (LASSO). In: The 8th annual basic science international conference: coverage of basic sciences toward the World’s Sustainability Challanges (2018).

[18] Newman AM, Steen CB, Liu CL, Gentles AJ, Chaudhuri AA, Scherer F (2019). Determining cell type abundance and expression from bulk tissues with digital cytometry. Nat Biotechnol.

[19] Zhao TX, Mallat Z (2019). Targeting the immune system in atherosclerosis: JACC state-of-the-art review. J Am Coll Cardiol.

[20] Moore KJ, Koplev S, Fisher EA, Tabas I, Bjorkegren JLM, Doran AC (2018). Macrophage trafficking, inflammatory resolution, and genomics in atherosclerosis: JACC macrophage in CVD series (Part 2). J Am Coll Cardiol.

[21] Zavaczki E, Gall T, Zarjou A, Hendrik Z, Potor L, Toth CZ (2020). Ferryl hemoglobin inhibits osteoclastic differentiation of macrophages in hemorrhaged atherosclerotic plaques. Oxid Med Cell Longev.

[22] Nie H, Yan C, Zhou W, Li T (2022). Analysis of immune and inflammation characteristics of atherosclerosis from different sample sources. Oxid Med Cell Longev.

[23] Liu C, Zhang H, Chen Y, Wang S, Chen Z, Liu Z (2020). Identifying RBM47, HCK, CD53, TYROBP, and HAVCR2 as hub genes in advanced atherosclerotic plaques by network-based analysis and validation. Front Genet.

[24] Lu J, Peng Y, Huang R, Feng Z, Fan Y, Wang H (2021). Elevated TYROBP expression predicts poor prognosis and high tumor immune infiltration in patients with low-grade glioma. BMC Cancer.

[25] Yuan S, Carter P, Bruzelius M, Vithayathil M, Kar S, Mason AM (2020). Effects of tumour necrosis factor on cardiovascular disease and cancer: a two-sample Mendelian randomization study. EBioMedicine.

[26] Bilgic Gazioglu S, Akan G, Atalar F, Erten G (2015). PAI-1 and TNF-alpha profiles of adipose tissue in obese cardiovascular disease patients. Int J Clin Exp Pathol.

[27] Wei Y, Zhu M, Corbalan-Campos J, Heyll K, Weber C, Schober A (2015). Regulation of Csf1r and Bcl6 in macrophages mediates the stage-specific effects of microRNA-155 on atherosclerosis. Arterioscler Thromb Vasc Biol.

[28] Lin CS, Hsieh PS, Hwang LL, Lee YH, Tsai SH, Tu YC (2018). The CCL5/CCR5 axis promotes vascular smooth muscle cell proliferation and atherogenic phenotype switching. Cell Physiol Biochem.

[29] Wang X, Han Y, Peng J, He J (2021). CCR5 is a prognostic biomarker and an immune regulator for triple negative breast cancer. Aging (Albany NY).

[30] Zhao B, Wang D, Liu Y, Zhang X, Wan Z, Wang J (2020). Six-gene signature associated with immune cells in the progression of atherosclerosis discovered by comprehensive bioinformatics analyses. Cardiovasc Ther.

[31] Xu X, Hao Y, Wu J, Zhao J, Xiong S (2021). Assessment of weighted gene co-expression network analysis to explore key pathways and novel biomarkers in muscular dystrophy. Pharmgenom Pers Med.

[32] Brawn LC, Hayward RD, Koronakis V (2007). Salmonella SPI1 effector SipA persists after entry and cooperates with a SPI2 effector to regulate phagosome maturation and intracellular replication. Cell Host Microbe.

[33] Salih MAM, Fakiola M, Lyons PA, Younis BM, Musa AM, Elhassan AM (2017). Expression profiling of Sudanese visceral leishmaniasis patients pre-and post-treatment with sodium stibogluconate. Parasite Immunol.

[34] Yao Z, Getting SJ, Locke IC (2021). Regulation of TNF-induced osteoclast differentiation. Cells.

[35] Vallejo A, Abad-Fernandez M, Moreno S, Moreno A, Perez-Elias MJ, Dronda F (2015). High levels of CD4(+) CTLA-4(+) Treg cells and CCR5 density in HIV-1-infected patients with visceral leishmaniasis. Eur J Clin Microbiol Infect Dis.

[36] Lee D, Shin KJ, Kim DW, Yoon KA, Choi YJ, Lee BNR (2018). CCL4 enhances preosteoclast migration and its receptor CCR5 downregulation by RANKL promotes osteoclastogenesis. Cell Death Dis.

[37] Li Z, Sun Y, He M, Liu J (2021). Differentially-expressed mRNAs, microRNAs and long noncoding RNAs in intervertebral disc degeneration identified by RNA-sequencing. Bioengineered.

[38] Skinkyte-Juskiene R, Kogelman LJA, Kadarmideen HN (2018). Transcription factor co-expression networks of adipose RNA-Seq data reveal regulatory mechanisms of obesity. Curr Genom.

[39] Hrdlicka HC, Lee SK, Delany AM (2019). MicroRNAs are critical regulators of osteoclast differentiation. Curr Mol Biol Rep.

[40] Szukiewicz D, Wojciechowska M, Bilska A, Stangret A, Szewczyk G, Mittal TK (2015). Aspirin action in endothelial cells: different patterns of response between chemokine CX3CL1/CX3CR1 and TNF-alpha/TNFR1 signaling pathways. Cardiovasc Drugs Ther.

[41] Li X, Ye Z, Guo Q, Wang E, Pan Y (2021). PDTC ameliorates neuropathic pain by inhibiting microglial activation via blockage of the TNFalpha-CX3CR1 pathway. Eur J Histochem.

[42] Morimura S, Oka T, Sugaya M, Sato S (2016). CX3CR1 deficiency attenuates imiquimod-induced psoriasis-like skin inflammation with decreased M1 macrophages. J Dermatol Sci.

[43] Garre JM, Silva HM, Lafaille JJ, Yang G (2017). CX3CR1(+) monocytes modulate learning and learning-dependent dendritic spine remodeling via TNF-alpha. Nat Med.

[44] Chistiakov DA, Sobenin IA, Orekhov AN (2013). Regulatory T cells in atherosclerosis and strategies to induce the endogenous atheroprotective immune response. Immunol Lett.

[45] Yang J, Liu R, Deng Y, Qian J, Lu Z, Wang Y (2017). MiR-15a/16 deficiency enhances anti-tumor immunity of glioma-infiltrating CD8+ T cells through targeting mTOR. Int J Cancer.

[46] Han N, Li X, Wang Y, Wang L, Zhang C, Zhang Z (2021). Increased tumor-infiltrating plasmacytoid dendritic cells promote cancer cell proliferation and invasion via TNF-alpha/NF-kappaB/CXCR-4 pathway in oral squamous cell carcinoma. J Cancer.

[47] Ni Y, Zhuge F, Ni L, Nagata N, Yamashita T, Mukaida N (2022). CX3CL1/CX3CR1 interaction protects against lipotoxicity-induced nonalcoholic steatohepatitis by regulating macrophage migration and M1/M2 status. Metabolism.

[48] Li B, Song X, Guo W, Hou Y, Hu H, Ge W (2021). Single-cell transcriptome profiles reveal fibrocytes as potential targets of cell therapies for abdominal aortic aneurysm. Front Cardiovasc Med.

