# Supplementary material for: Identification of key genes and pathways in atherosclerosis using integrated bioinformatics analysis
Source: BMC Med Genomics. 2023 May 13;16:102. doi: 10.1186/s12920-023-01533-8 (PMC10183119; doi:10.1186/s12920-023-01533-8)
Supplement: Supplementary file 1 — Additional file 1. Table S1. The KEGG pathway of Module 1, Related to Figure 4A. Table S2. The KEGG pathway of Module 2, Related to Figure 4B. Table S3. The KEGG pathway of Module 3, Related to Figure 4C. [file 12920_2023_1533_MOESM1_ESM.docx]

**Additional file 1 The KEGG pathway analysis of the top 3 Modules**

Table S1 The KEGG pathway of Module 1, Related to Figure 4A

| **KEGG pathway** | **Description** | **p-value** | **Count** |
| --- | --- | --- | --- |
| hsa04061 | Viral protein interaction with cytokine and receptor | 0.000 | 11 |
| hsa04060 | Cytokine-cytokine receptor interaction | 0.000 | 13 |
| hsa05133 | Pertussis | 0.000 | 7 |
| hsa04062 | Chemokine signaling pathway | 0.000 | 9 |
| hsa05142 | Chagas disease (American trypanosomiasis) | 0.000 | 7 |
| hsa05152 | Tuberculosis | 0.000 | 8 |
| hsa05163 | Human cytomegalovirus infection | 0.000 | 8 |
| hsa05134 | Legionellosis | 0.000 | 5 |
| hsa05020 | Prion diseases | 0.000 | 4 |
| *hsa05140* | ***Leishmaniasis*** | 0.000 | 5 |
| hsa04610 | Complement and coagulation cascades | 0.000 | 5 |
| hsa04620 | Toll-like receptor signaling pathway | 0.000 | 5 |
| *hsa04380* | ***Osteoclast differentiation*** | 0.001 | 5 |
| hsa05323 | Rheumatoid arthritis | 0.002 | 4 |
| hsa05150 | Staphylococcus aureus infection | 0.003 | 4 |
| *hsa04640* | ***Hematopoietic cell lineage*** | 0.003 | 4 |
| hsa04668 | TNF signaling pathway | 0.004 | 4 |
| hsa04670 | Leukocyte transendothelial migration | 0.004 | 4 |
| hsa05322 | Systemic lupus erythematosus | 0.007 | 4 |
| hsa04623 | Cytosolic DNA-sensing pathway | 0.007 | 3 |
| hsa05418 | Fluid shear stress and atherosclerosis | 0.007 | 4 |
| *hsa04145* | ***Phagosome*** | 0.010 | 4 |
| hsa05132 | Salmonella infection | 0.013 | 3 |
| hsa05164 | Influenza A | 0.013 | 4 |
| hsa04621 | NOD-like receptor signaling pathway | 0.016 | 4 |
| hsa04657 | IL-17 signaling pathway | 0.017 | 3 |
| hsa01523 | Antifolate resistance | 0.020 | 2 |
| hsa05146 | Amoebiasis | 0.021 | 3 |
| hsa04625 | C-type lectin receptor signaling pathway | 0.021 | 3 |
| hsa05145 | Toxoplasmosis | 0.025 | 3 |
| hsa05143 | African trypanosomiasis | 0.025 | 2 |
| hsa05135 | Yersinia infection | 0.029 | 3 |
| hsa05332 | Graft-versus-host disease | 0.029 | 2 |
| hsa04940 | Type I diabetes mellitus | 0.031 | 2 |
| hsa05144 | Malaria | 0.038 | 2 |
| hsa05014 | Amyotrophic lateral sclerosis (ALS) | 0.040 | 2 |
| hsa04514 | Cell adhesion molecules (CAMs) | 0.043 | 3 |

Table S2 The KEGG pathway of Module 2, Related to Figure 4B

| **KEGG pathway** | **Description** | **p-value** | **Count** |
| --- | --- | --- | --- |
| *hsa05140* | ***Leishmaniasis*** | 0.000 | 11 |
| hsa04612 | Antigen processing and presentation | 0.000 | 11 |
| hsa05150 | Staphylococcus aureus infection | 0.000 | 11 |
| hsa05152 | Tuberculosis | 0.000 | 13 |
| *hsa04145* | ***Phagosome*** | 0.000 | 12 |
| hsa05169 | Epstein-Barr virus infection | 0.000 | 12 |
| hsa05310 | Asthma | 0.000 | 7 |
| hsa05416 | Viral myocarditis | 0.000 | 8 |
| hsa05323 | Rheumatoid arthritis | 0.000 | 9 |
| hsa05322 | Systemic lupus erythematosus | 0.000 | 10 |
| hsa05330 | Allograft rejection | 0.000 | 7 |
| *hsa04640* | ***Hematopoietic cell lineage*** | 0.000 | 9 |
| hsa05321 | Inflammatory bowel disease (IBD) | 0.000 | 8 |
| hsa05332 | Graft-versus-host disease | 0.000 | 7 |
| hsa04940 | Type I diabetes mellitus | 0.000 | 7 |
| hsa04672 | Intestinal immune network for IgA production | 0.000 | 7 |
| hsa05320 | Autoimmune thyroid disease | 0.000 | 7 |
| hsa05145 | Toxoplasmosis | 0.000 | 8 |
| hsa05164 | Influenza A | 0.000 | 9 |
| hsa04658 | Th1 and Th2 cell differentiation | 0.000 | 7 |
| hsa04514 | Cell adhesion molecules (CAMs) | 0.000 | 8 |
| hsa05166 | Human T-cell leukemia virus 1 infection | 0.000 | 9 |
| hsa04659 | Th17 cell differentiation | 0.000 | 7 |
| hsa04620 | Toll-like receptor signaling pathway | 0.000 | 6 |
| hsa05168 | Herpes simplex virus 1 infection | 0.000 | 10 |
| hsa04064 | NF-kappa B signaling pathway | 0.000 | 5 |
| *hsa04380* | ***Osteoclast differentiation*** | 0.001 | 5 |
| hsa05133 | Pertussis | 0.013 | 3 |
| hsa04666 | Fc gamma R-mediated phagocytosis | 0.022 | 3 |
| hsa04621 | NOD-like receptor signaling pathway | 0.022 | 4 |
| hsa05340 | Primary immunodeficiency | 0.032 | 2 |
| hsa04142 | Lysosome | 0.042 | 3 |
| hsa04611 | Platelet activation | 0.042 | 3 |
| hsa05144 | Malaria | 0.046 | 2 |
| hsa04650 | Natural killer cell mediated cytotoxicity | 0.046 | 3 |

Table S3 The KEGG pathway of Module 3, Related to Figure 4C

| KEGG pathway | Description | p-value | Count |
| --- | --- | --- | --- |
| *hsa04145* | ***Phagosome*** | 0.000 | 7 |
| *hsa04380* | ***Osteoclast differentiation*** | 0.002 | 5 |
| hsa04664 | Fc epsilon RI signaling pathway | 0.002 | 4 |
| *hsa05140* | ***Leishmaniasis*** | 0.002 | 4 |
| hsa04662 | B cell receptor signaling pathway | 0.002 | 4 |
| *hsa04640* | ***Hematopoietic cell lineage*** | 0.004 | 4 |
| hsa04660 | T cell receptor signaling pathway | 0.004 | 4 |
| hsa04611 | Platelet activation | 0.007 | 4 |
| hsa04650 | Natural killer cell mediated cytotoxicity | 0.008 | 4 |
| hsa04658 | Th1 and Th2 cell differentiation | 0.027 | 3 |
| hsa04666 | Fc gamma R-mediated phagocytosis | 0.027 | 3 |
| hsa04659 | Th17 cell differentiation | 0.037 | 3 |
| hsa04670 | Leukocyte transendothelial migration | 0.039 | 3 |
| hsa05340 | Primary immunodeficiency | 0.043 | 2 |
